# Supplementary material for: Multiple social encounters can eliminate Crozier’s paradox and stabilise genetic kin recognition
Source: Nat Commun. 2022 Jul 6;13:3902. doi: 10.1038/s41467-022-31545-4 (PMC9259605; doi:10.1038/s41467-022-31545-4)
Supplement: Supplementary file 1 — Supplementary Information [file 41467_2022_31545_MOESM1_ESM.pdf]

Supplementary Materials for

Multiple social encounters can eliminate Crozier's paradox and stabilise genetic kin recognition

Thomas W Scott, Alan Grafen, Stuart A West

Correspondence to: thomas.scott@zoo.ox.ac.uk

This file includes:

- Supplementary Figures 1 to 8.
- Supplementary Discussion 1 to 3.
- Supplementary References.

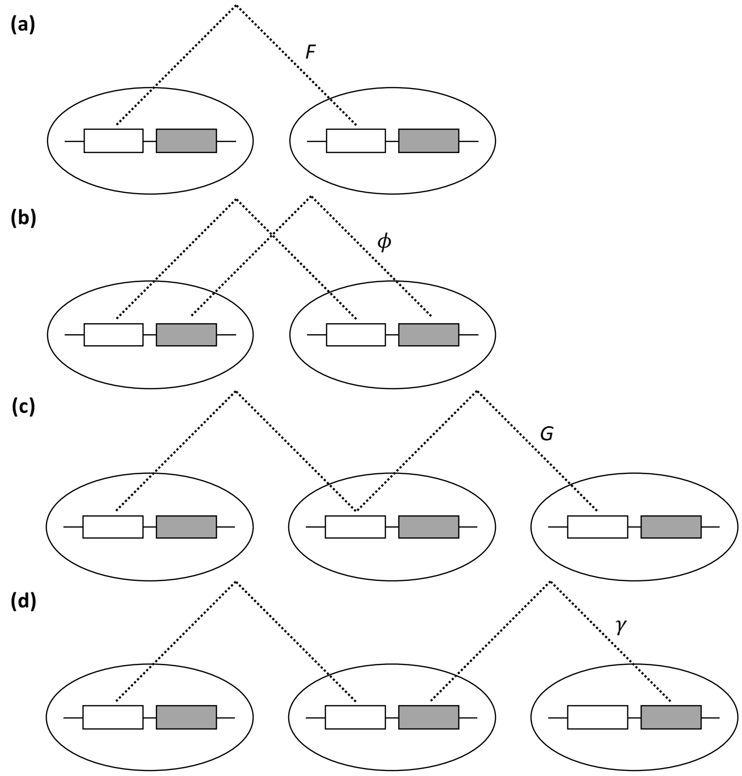

Supplementary Figure 1. Probabilities of being identical by descent (IBD). Individuals are represented by oval boxes. Loci are represented by rectangles (empty and grey rectangles represent two different loci). Individuals are drawn from the same deme. In parts A and B, there are two individuals, and these are drawn without replacement. In parts C and D, there are three individuals, the first two drawn without replacement, and the third individual drawn with replacement of the first two. Dotted lines connect gene copies that are IBD (coalesce in finite time / coalesce in the same deme). F gives the probability that two individuals are IBD at a given (single) locus. Φ gives the probability that two individuals are IBD at two given loci. G gives the probability that three individuals are IBD at a given (single) locus. γ gives the probability that individuals 1 and 2 are IBD at one locus, and individuals 2 and 3 are IBD at a second locus. This figure reconstructs and extends (adds part C to) a figure in Rousset & Roze[1] (henceforth R&R).

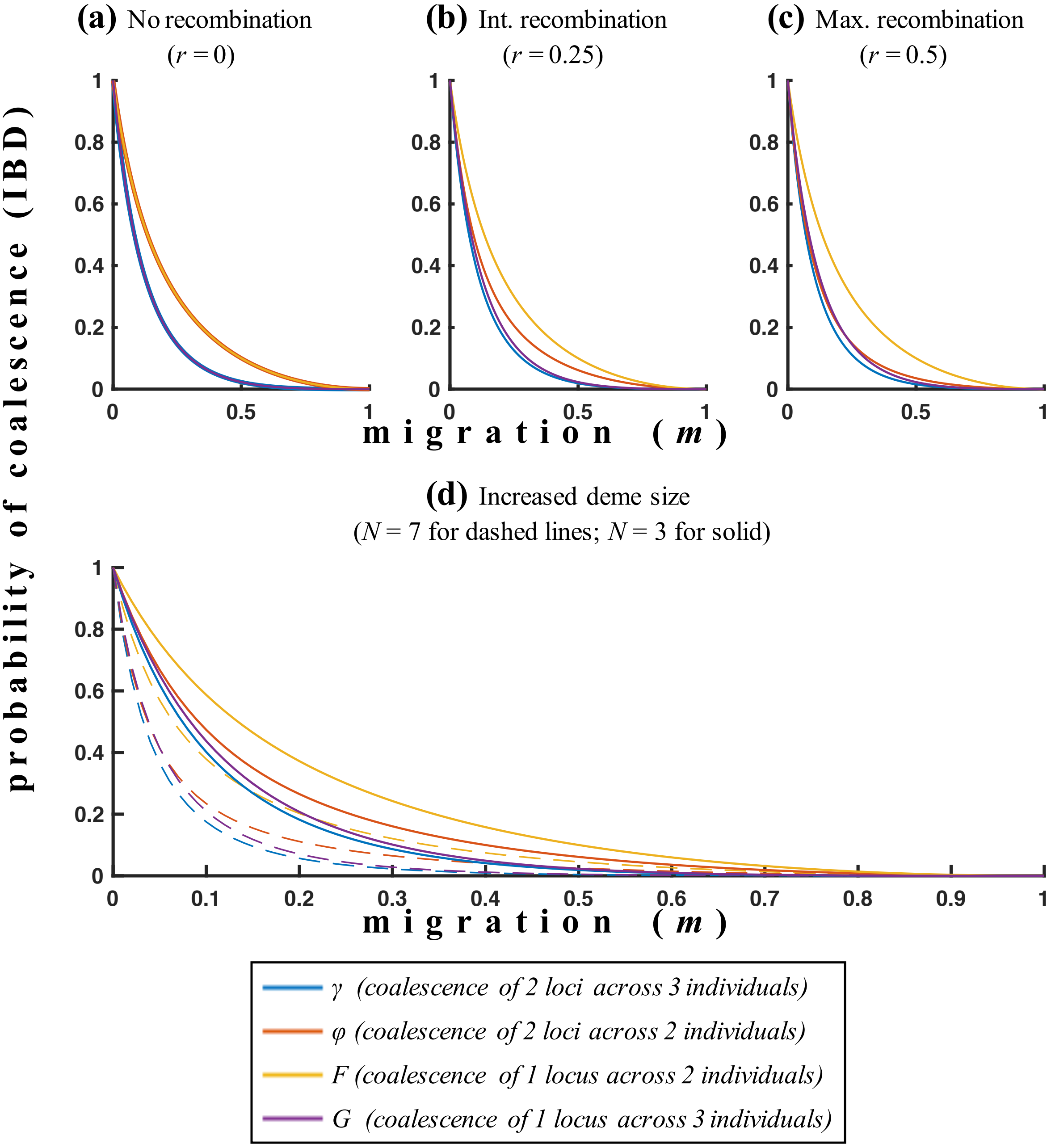

Supplementary Figure 2. Coalescence probabilities (*F*,*φ*,*γ*,*G*) plotted for different demographic parameter values (*N*,*r*,*m*). Recombination is varied across panels a–d (r=0 in panel a; r=0.25 in panels b & d; r=0.5 in panel c). Deme size is set to N=3 in panels a–c, and is varied in panel d. Migration rate (m) is plotted on the x axes, and coalescence probabilities (F,φ,γ,G) are plotted on the y axes.

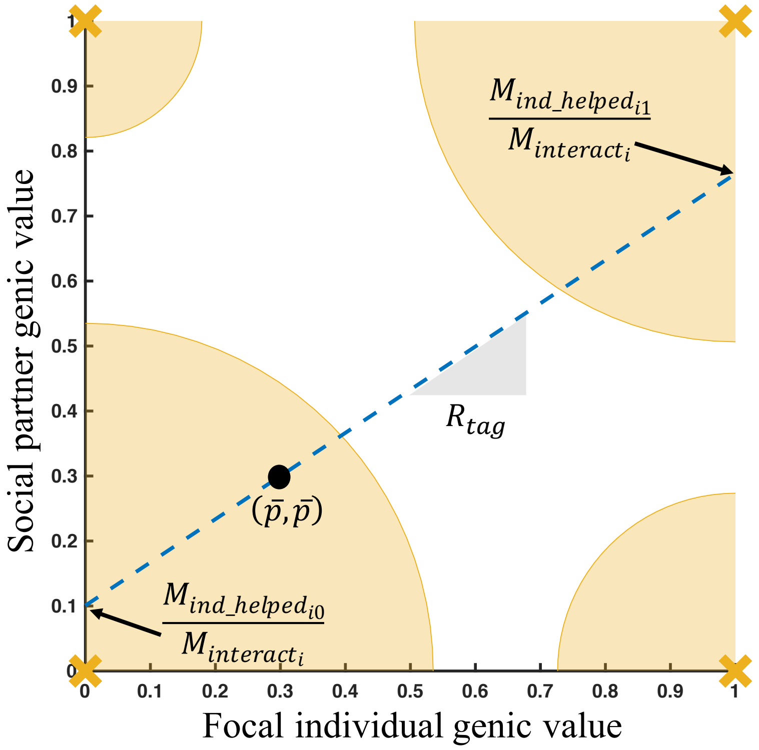

Supplementary Figure 3. Interpretation of relatedness (R_{tag}) as a line of best fit to a regression of actor's genic value on social partner's genic value. For all possible scenarios that may arise, the genic (trait) value of a focal individual is plotted (orange crosses) alongside the genic value of a social partner. Individuals have genic values of one (conditional helpers) or zero (defectors). Therefore, all data points (orange crosses) are confined to the extremes of the x and y axes, which range from zero to one. The density of data points at each axis-extreme, corresponding to the likelihood of a particular type of interaction occurring, is represented by the area of the orange quarter-circles. Relatedness (R_{tag}) is then given by the gradient of the regression line through these data points, where this regression line is forced through the population mean genic value $(\bar{p}, \bar{p})$ (blue dotted line). This figure was generated with the parameter values m=0.1, r=0.1, N=10, x_i=0.05, p_i=0.3 & $\bar{p}$=0.3, leading to a relatedness between an actor and its social partner of R_{tag}=0.6642.

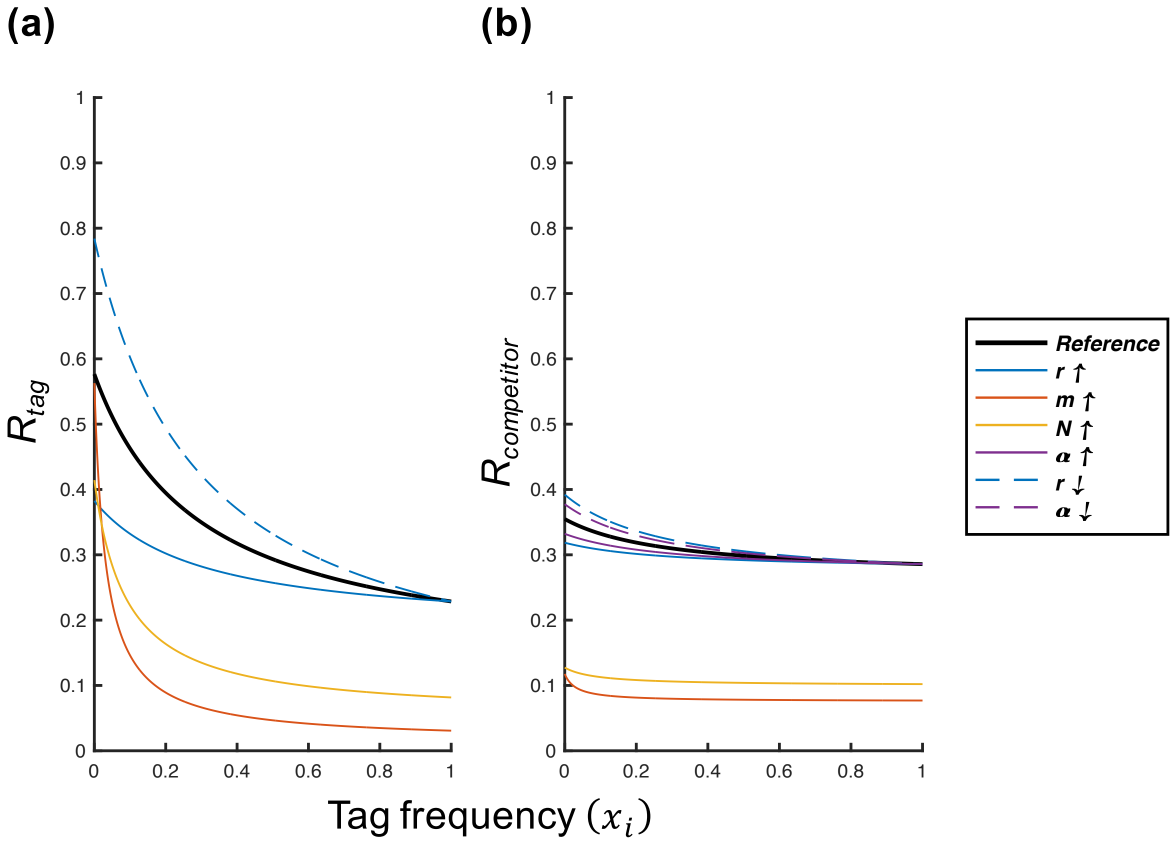

Supplementary Figure 4. Relatedness coefficients as functions of model parameters. Panel a plots relatedness to social partners (R_{tag}), and panel b plots relatedness to competitors ($R_{competitor}$). Relatedness coefficients are plotted against tag frequency (x_i). Relatedness coefficients increase as tag frequency (x_i) decreases (tag becomes a more reliable indicator of kinship), especially with reduced recombination (r) between tag and trait (panels a & b). Increased migration (m) and deme size (N) leads to reduced relatedness (panels a & b). Partner search (α) has no effect on relatedness to social partners (R_{tag}; panel a), but decreases relatedness to local competitors ($R_{competitor}$; panel b).

The following parameter values were assumed in the reference cases (black lines): m=0.2, r=0.25, N=6, α=0.5; the other cases (coloured lines) manipulated one parameter whilst keeping all the other reference parameters unchanged, such that: r=0.5 (blue lines), m=0.6 (red lines), N=20 (yellow lines), α=1 (purple lines), r=0 (blue dotted lines), α=0 (purple dotted lines). These plots assumed that helpers are evenly distributed across tags (no linkage disequilibrium), such that $p_i = \bar{p}$.

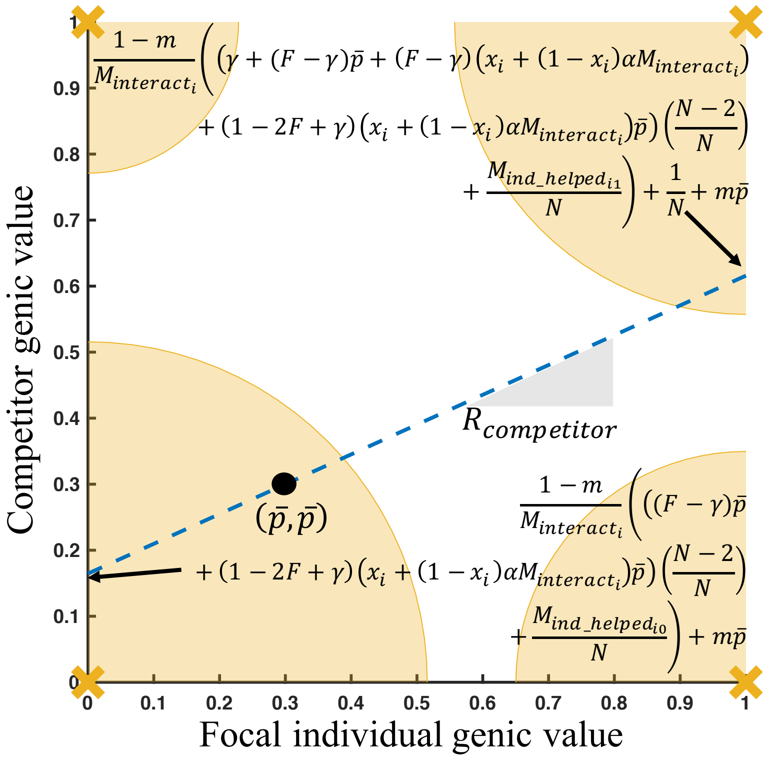

Supplementary Figure 5. Interpretation of relatedness (*$R_{competitor}$*) as a line of best fit to a regression of actor's genic value on local competitor's genic value. For all possible scenarios that may arise, the genic (trait) value of a focal individual is plotted (orange crosses) alongside the genic value of a competitor. Individuals have genic values of one (conditional helpers) or zero (defectors). Therefore, all data points (orange crosses) are confined to the extremes of the *x* and *y* axes, which range from zero to one. The density of data points at each axis-extreme, corresponding to the likelihood of a given scenario, is represented by the area of the orange quarter-circles. Relatedness ($R_{competitor}$) is then given by the gradient of the regression line through these data points, where this regression line is forced through the population mean genic value $(\bar{p},\bar{p})$ (blue dotted line). This figure was generated with the parameter values m=0.1, r=0.1, N=10, x_i=0.05, p_i=0.3, $\bar{p}$=0.3 & α=0.4 (same as Supplementary Figure 3), leading to a relatedness between an actor and its local competitors of $R_{competitor}$=0.4062.

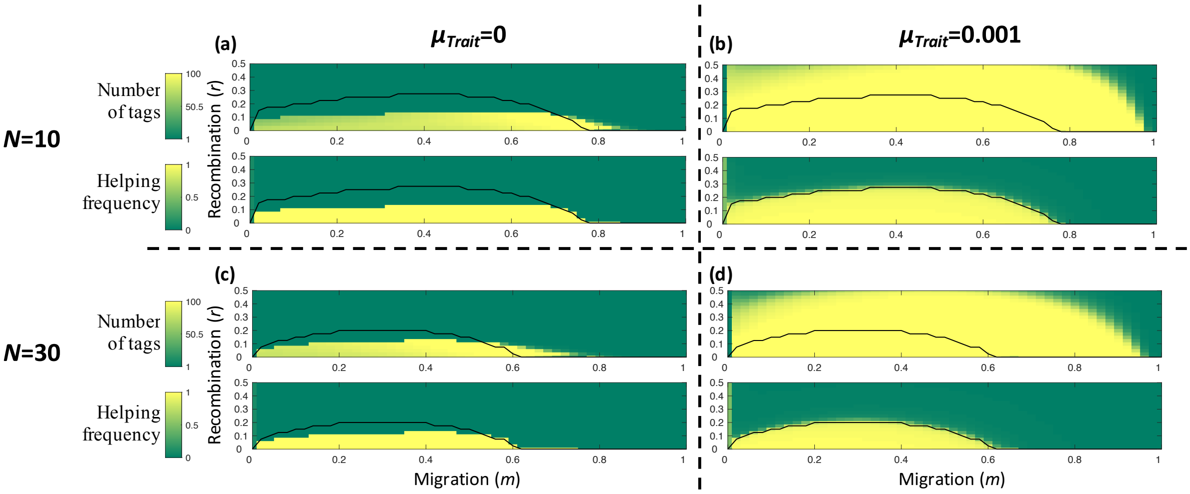

Supplementary Figure 6. Equilibrium tag diversity and helper frequency when partner search is uncostly and unrestricted (α=1 & c_{search}=0) – *population genetic model.* The population genetic model (Equations 2-16) was numerically solved for different: migration rates (*m*; *x axes*); recombination rates (*r*; *y axes*); deme sizes (*N*; upper vs lower quadrants); trait mutation rates (μ_{trait}; left vs right quadrants). For each trial, equilibrium number of segregating tags, *L** (upper graph within each quadrant), and equilibrium frequency of the conditional helping allele (lower graph within each quadrant) are plotted. The region underneath the black lines correspond to when inclusive fitness theory predicts that genetic kin recognition may evolve (Equation 25 is satisfied, meaning kin discrimination is favoured).

We find that, when there is trait mutation (b and d), there is a perfect correspondence between the *predicted* (black lines) and *actual* region of parameter space where genetic kin recognition evolves. When there is no trait mutation, indiscriminate defection evolves more often than is predicted by Equation 25 (some green underneath the black lines in the helper frequency graphs in a and c). We see that tag diversity is sometimes maintained even when altruistic helping does not evolve (some yellow above the black lines in the tag diversity graphs). This is either because helpers have been purged, leading to neutral evolution at the tag locus (a and c), or because helpers exist at low frequency (mutation-selection balance), incentivising kin recognition, and meaning tag diversity is maintained by selection (b and d), even though altruistic helping is negatively selected.

This plot was generated using: the parameter values *b*=0.3, *c*=0.1, L_{max}=100, α=1, c_{search}=0, μ_{tag}=0; all parameter combinations of *m*=0,0.02,0.04,…,1 & *r*=0,0.025,0.05,…,0.5.

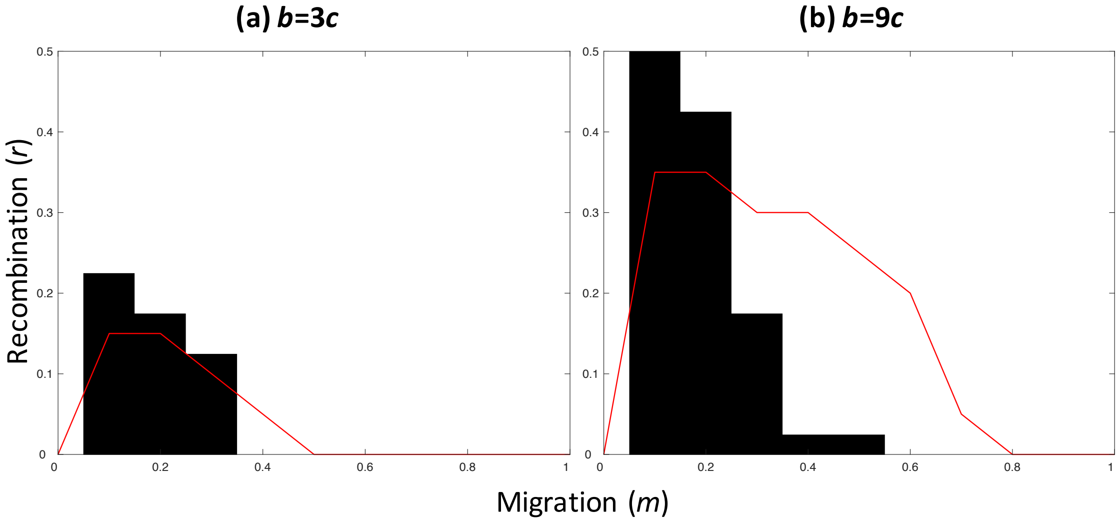

Supplementary Figure 7. The evolution of kin discrimination based on genetic cues when partner search is uncostly and unrestrained (α=1, c_{search}=0) – *agent-based simulation.* A population of 501 individuals were implemented in an agent-based simulation over 5000 generations, for different: migration rates (*m*; *x axes*); recombination rates (*r*; *y axes*); benefit of helping (a vs b). Black shading shows where kin discrimination based on genetic cues evolved (helper frequency, *coop*>0.3; number of tags, *L*>1.1; *coop* & *L* are taken as averages over the final 3750 generations). The area below the red lines show where Equation 25 / 1 is satisfied, meaning kin discrimination optimises inclusive fitness. We find that kin discrimination based on genetic cues evolves in this agent-based simulation. There is not a complete correspondence between the black shaded region (kin discrimination based on genetic cues present) and the area under the red line (kin discrimination based on genetic cues expected), but this is to be expected, given that our expected region (red line) was derived under the assumptions of large deme size (*N*), weak selection, and an infinite population, all of which are violated in this agent-based simulation. This plot was generated using: the parameters *N*=3; *c*=0.3, L_{max}=10, α=1, μ_{tag}=0.005, μ_{trait}=0.005, c_{search}=0; all parameter combinations of *m*=0,0.1,0.2,…,1 & *r*=0,0.05,0.1,…,0.5.

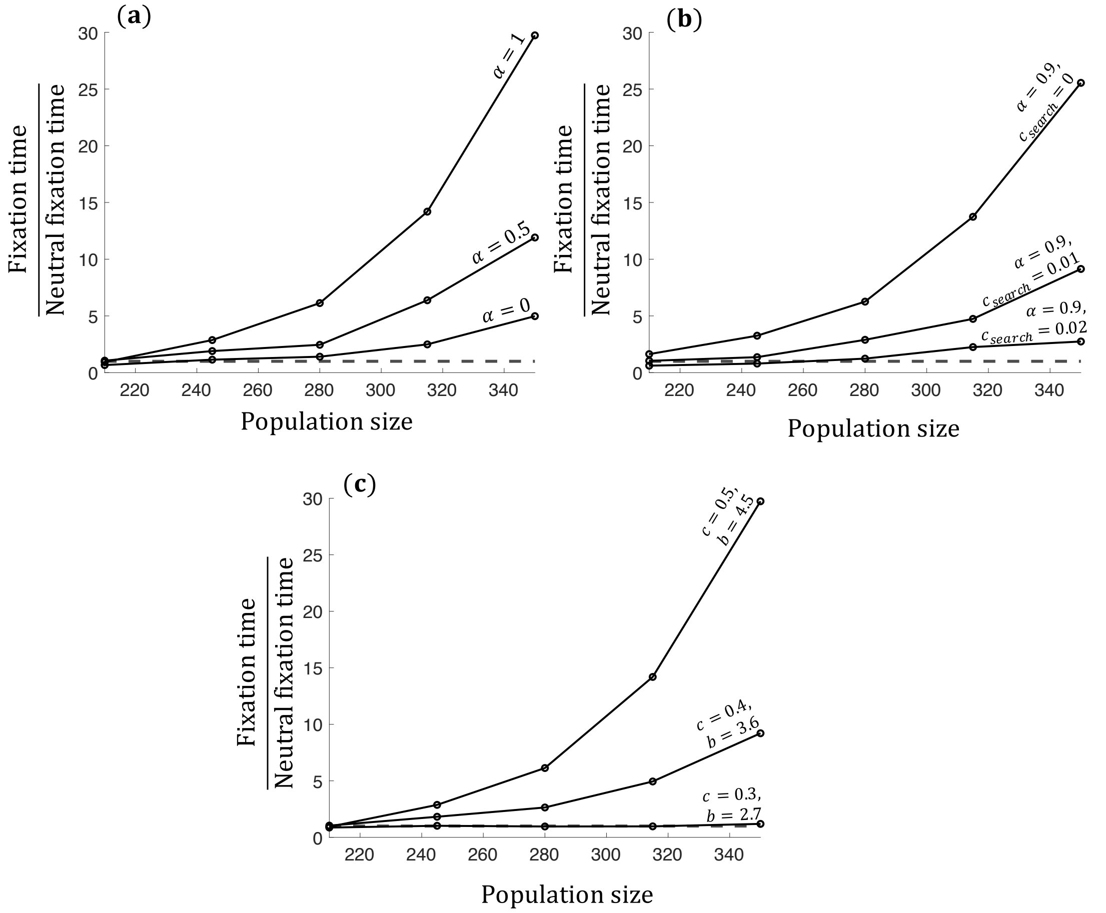

Supplementary Figure 8. Demonstration of balancing selection in the finite population model (agent-based simulation). Finite populations of individuals of a given size were implemented in a version of the agent-based simulation model in which there is no tag mutation (μ_{tag}=0). For each trial, the time taken for tag diversity to be lost (fixation time) is recorded and divided by the time taken for tag diversity to be lost in the corresponding neutral scenario (neutral fixation time). This *tag fixation ratio* is taken as an average over many trials, and plotted against population size. Balancing selection is present if the ratio of fixation times is: (*i*) greater than 1 for all population sizes, and (*ii*) increasing with population size (with a steeper increase indicative of stronger balancing selection). We assume the following parameter values, except where labelled otherwise: c=0.5, b=4.5, α=1, *N*=7, L_{max}=2, μ_{trait}=0.005, *m*=0.01, *r*=0.01. We see that balancing selection can operate under conditions of low migration and recombination even in the absence of partner search (panel a, α=0 line). Balancing selection is strengthened by increasing partner search (panel a), reducing the cost of partner search (panel b), and increasing the magnitude of the social payoffs *b* and *c* (panel c).

Supplementary Discussion 1: Relation to previous theory.

Our results build on previous population genetic models of genetic kin recognition, which did not permit partner search, so were implicitly considering the α=0 case. Previous studies have analysed the same island lifecycle as ours[1-3], as well as different lifecycles[4-9]. Each of these studies found that selection arising from social behaviour alone (i.e. *b*, *c*) was insufficient to give rare tags an advantage and allow tag diversity to be maintained. An exception to this general conclusion is that, in R&R's treatment, when recombination and migration are low, and selection on social behaviour (*b*,*c*) is strong, two tags (but no more than two) could be maintained alongside relatively low frequencies of the conditional helping allele, resulting in a form of kin discrimination, albeit one that is not very precise (due to the low number of recognition tags) or cooperative (due to the low frequency of the conditional helping allele).

Aside from the rare exception noted by R&R, these previous theoretical studies therefore recovered Crozier's paradox. Tag diversity could only persist in these models in the presence of rapid tag mutation. Under restrictive conditions, where selection was strong (e.g. high *b* & *c*) and tag-trait linkage was tight (low *r*), rapid tag mutation could overwhelm selection against tag diversity, allowing some (finite amount of) tag diversity to be maintained alongside some low / intermediate frequency of the conditional helping allele. The resulting kin discrimination was accordingly not very precise or cooperative (though precision may increase with tag mutation rate).

The key difference in our model is that, by allowing for partner search (α>0), we found that kin discrimination based on genetic cues evolves to be stable under a broad, permissive parameter space (i.e. for a greater range of migration & recombination rates). In addition, we found that selection alone maintains tag diversity (no tag mutation), leading to arbitrarily high amounts of tag diversity (i.e. all L_{max} tags are maintained), with the conditional helping allele going to fixation. The resulting kin discrimination was highly precise and cooperative. Furthermore, our results still tend to hold in the case where selection on social behaviour (*b*,*c*) is weak. Previous results, as well as requiring sufficient tag mutation, also required selection on social behaviour (*b*,*c*) to be strong. Our results don't require strong selection, which again increases their generality.

It should be noted that, although previous theoretical studies found support for the hypothesis that selection on social behaviour alone (i.e. *b*, *c*) is insufficient to give an advantage for rare tags and allow tag diversity to be maintained (Crozier's paradox), many of these studies also found support for Crozier's resolution to the paradox. Crozier's resolution to the paradox was that rare tags could gain an advantage, stabilising tag diversity, if tags have some other function unrelated to their role in social behaviour, and rare tags are better than common tags for this unrelated function. R&R and Holman et al. (2013)[10] showed that such *extrinsic balancing selection* can indeed stabilise tag diversity. One way in which extrinsic balancing selection may arise is if recognition alleles have a role in parasite resistance[11]. This could favour rare alleles because they are less exploited by parasites. Another way in which extrinsic balancing selection may arise is if recognition alleles have a role in mate-choice[10]. This could favour rare alleles because using tag-matching as a way to avoid inbreeding is more effective for individuals that have rare tags.

Extrinsic balancing selection could therefore provide another mechanism for maintaining tag diversity and supporting kin discrimination, in addition to our mechanism, in which kin discrimination is supported by selection arising from the social behaviour alone (*b*,*c*). These mechanisms need not be mutually exclusive. However, one key thing to note here is that the extrinsic balancing selection solution is incomplete, because no one has yet given an account of why individuals should "choose", as recognition loci, those loci that are already under extrinsic balancing selection[12]. For instance, it is conceivable that an individual might

evolve to stop using a locus under extrinsic balancing selection as a recognition locus, and instead use a locus under no external selective forces. Doing so may often be favourable, as it may allow individuals to adopt social strategies (indiscriminate helping, conditional helping, defection) that are most appropriate to the situation (as revealed by Hamilton's Rule; Equation 25 / 1), rather than being forced to exhibit conditional helping in all cases, even when it is unfavourable. But in lieu of a theoretical examination of the evolution of 'choice of recognition locus', the extrinsic balancing selection hypothesis may provide another way for tag diversity to be maintained, stabilising kin discrimination.

Tag-based harming, as opposed to tag-based altruistic helping, has been examined elsewhere[2].

Supplementary Discussion 2: Conservative modelling assumptions.

From some perspectives, we have modelled an unfavourable scenario for genetic kin recognition and so, from these perspectives, our finding that it can be stable may be conservative. First, we assumed that social interactions are asymmetrical (one actor; one recipient), and that every time an individual abandons its social partner for a new social encounter, it can draw any individual (other than itself) from its interaction group. On this framework, the probability of encountering a given partner (e.g. a tag-matched partner) remains constant, regardless of how many times the focal individual has already re-associated for a new social encounter (how far along the social search it is).

We alternatively could have assumed that social interactions are reciprocal, with both individuals in the pair having the opportunity to provide as well as receive help. In this alternative framework, individuals would interact (reciprocally) a maximum of once per generation, with just one individual. Specifically, individuals would aggregate themselves into physical pairs. Tag-matched pairs would then socially interact, and tag-mismatched pairs would either forego the social interaction (1-α), or abandon their partner for a new social encounter (α). However, in this alternative framework, individuals that abandon their social partner would only have access to a subset of the interaction group (those that have not yet interacted) for further social encounters.

The important upshot of this is that, in this alternative framework, common tags will pair up and interact quickly (requiring few social encounters), meaning common tags will be removed disproportionately quickly from the pool of individuals that are available for social encounters. As a result, in this alternative framework, the interaction rate advantage for common tags will be less stark, favouring genetic kin recognition, relative to our (conservative) framework.

The second reason why our finding that genetic kin recognition can be stable is likely to be conservative is that we analysed Wright's island model, which is unfavourable to the evolution of altruism, because there is extreme local competition for resources (kin competition). For instance, in this island model, local competition is so strong that indiscriminate altruistic helping cannot evolve[1,13-16]. Other lifecycles, where local competition (kin competition) is less stark, are likely to lead to the evolution of discriminating altruism (genetic kin recognition) more permissively.

Third, we assumed that there is initially negligible tag diversity, and that there is no tag mutation, both of which increase the likelihood that rare tags are lost before they can acquire helpers. This allowed us to examine the evolution from scratch (as well as the maintenance) of kin recognition. However, we alternatively could have assumed that, initially, multiple tags segregate in the population, and they mutate, which may have been more favourable to the maintenance of genetic kin recognition.

Fourth, we modelled conditional helping as a discrete (all or nothing) trait. We could have alternatively (and more realistically, for animals) modelled conditional helping as a quantitative (variable) trait, where different individuals vary in their exact quantitative level of altruistic investment. A potentially important difference here is that, in the discrete scenario, cheating can (if negatively selected, and in the absence of trait mutation) be completely purged by selection. Once the risk of being cheated in social interactions has been eliminated, there is no longer any evolutionary incentive to restrict social interactions to kin. This halts selection for further tag diversification, and places an upper limit on the precision of kin recognition. In the alternative scenario, where helping is a quantitative trait, different individuals will cheat to different extents (due to trait variation), even in the evolutionary long term. This persistent low-level cheating may incentivise kin discrimination, relative to our scenario.

Finally, we focused on partner search, but alternative mechanisms could also potentially eliminate Crozier's paradox. These could include large synergistic costs to being cheated (sucker's payoff), or additional fitness costs of interacting with tag-matched individuals who turn out to be genealogical non-kin[17,18].

Supplementary Discussion 3: The importance of kinship (pedigree) relatedness.

Relatedness between social partners can be achieved in two main ways. The first is kinship (common ancestry). If two individuals share a common ancestor (e.g. grandmother), then they are likely to have inherited similar genes, and therefore be genetically similar (related). Field biologists can often infer kinship relatedness simply by observing an animal's family tree (pedigree).

The second way to achieve relatedness is by gene-matching. Kin-discriminating animals find their social partners by matching tags. A consequence of tag matching is that relatedness will be maximised (=1) at the tag locus. This is simply because any individuals that use tag-matching as a basis for social interaction will (trivially) be genetically identical at the locus responsible for encoding the tag. Furthermore, any loci that are in linkage disequilibrium with the tag locus may have heightened relatedness. This includes loci that are physically linked to the tag locus (i.e. on the same chromosome), as well as other loci, like the trait locus, that are associated with the tag locus due to co-adaptation.

The key difference between kinship and gene-matching, as causes of relatedness, is that kinship increases genetic similarity (relatedness) at all loci, whereas gene-matching only increases genetic similarity at a subsection of loci within the genome (the tag locus and those in linkage disequilibrium with it). This leads to the question – what type of relatedness is important for the evolution of discriminating altruism? Does discriminating altruism evolve in accordance with kinship (pedigree) relatedness, or tag locus relatedness, or something else?

The first thing to note here is that the evolution of *any* social trait will technically proceed in accordance with the relatedness measured at the locus (or loci) responsible for encoding the trait[19,20]. In the context of our model, evolution proceeds in accordance with relatedness at the trait locus. That is, only in generations where Equation 25 / 1 is satisfied, when R_{tag} and $R_{competitor}$ are measured at the trait locus, will the conditional helping allele increase in frequency. As we said before, trait locus relatedness may deviate from kinship (pedigree) relatedness due to tag-trait linkage disequilibrium.

This might seem to imply that knowledge about kinship (pedigree) relatedness is insufficient for determining the outcome of social evolution. However, one important result of our analysis is that, when partner search is abundant (high α) and cheap (low c_{search}), and kin discrimination evolves, tags evolve to obtain approximately equal frequencies and cheater loads, meaning there is no linkage disequilibrium at equilibrium. This means that trait locus relatedness converges on kinship (pedigree) relatedness in the evolutionary long term (Figure 2c blue line). Therefore, it is kinship (pedigree) relatedness that matters when we are thinking about evolutionarily stable social traits, as predicted by Hamilton and Grafen[17,19,21].

This means we can interpret the coefficients of relatedness in Equation 25 / 1 as coefficients of kinship (pedigree) relatedness. This is good news for empiricists, who can calculate, for natural populations, the coefficients of relatedness that feature in Equation 25 / 1, without having to know genetic details like linkage disequilibrium. It also implies that 'greenbeard effects', where relatedness at specific loci deviate from kinship (pedigree) relatedness, are generally unimportant for the evolution of kin discrimination based on genetic cues. An exception arises under some restrictive conditions when partner search (α) is low, where low levels of discriminating altruism may evolve despite low pedigree relatedness, through 'beard chromodynamics'[5].

Supplementary References

1. Rousset, F. & Roze, D. Constraints on the origin and maintenance of genetic kin recognition. *Evolution* **61,** 2320–2330 (2007).
2. Lehmann, L., Feldman, M. W. & Rousset, F. On the evolution of harming and recognition in finite panmictic and infinite structured populations. *Evolution* **63,** 2896–2913 (2009).
3. Gardner, A. & West, S. A. Social Evolution: The Decline and Fall of Genetic Kin Recognition. *Current Biology* **17,** R810–R812 (2007).
4. Traulsen, A. & Nowak, M. A. Chromodynamics of cooperation in finite populations. *PLoS ONE* **2,** e270 (2007).
5. Jansen, V. A. A. & van Baalen, M. Altruism through beard chromodynamics. *Nature* **440,** 663–666 (2006).
6. Antal, T., Ohtsuki, H., Wakeley, J., Taylor, P. D. & Nowak, M. A. Evolution of cooperation by phenotypic similarity. *Proc. Natl. Acad. Sci. U.S.A.* **106,** 8597–8600 (2009).
7. Axelrod, R., Hammond, R. A. & Grafen, A. Altruism via kin-selection strategies that rely on arbitrary tags with which they coevolve. *Evolution* **58,** 1833–1838 (2004).
8. Kroumi, D. & Lessard, S. Evolution of cooperation in a multidimensional phenotype space. *Theoretical Population Biology* **102,** 60–75 (2015).
9. Traulsen, A. Mechanisms for similarity based cooperation. *The European Physical Journal B* **63,** 363–371 (2008).
10. Holman, L., van Zweden, J. S., Linksvayer, T. A., d'Ettorre, P. & d'Ettorre, P. Crozier's paradox revisited: maintenance of genetic recognition systems by disassortative mating. *BMC Evol Biol* **13,** 211–13 (2013).
11. Crozier, R. H. Genetic clonal recognition abilities in marine invertebrates must be maintained by selection for something else. *Evolution* **40,** 1100–1101 (1986).
12. Hammerstein, P. Darwinian adaptation, population genetics and the streetcar theory of evolution. *Journal of Mathematical Biology* **34,** 511–532 (1996).
13. Taylor, P. D. Altruism in viscous populations — an inclusive fitness model. *Evol Ecol* **6,** 352–356 (1992).
14. Frank, S. A. *Foundations of Social Evolution*. (Princeton University Press, 1998). doi:10.1038/sj.hdy.6885351
15. Rousset, F. *Genetic Structure and Selection in Subdivided Populations (MPB-40)*. (Princeton University Press, 2004).
16. Taylor, P. D. Inclusive Fitness in a Homogeneous Environment. *Proceedings: Biological Sciences* **249,** 299–302 (1992).
17. Grafen, A. Do animals really recognize kin? *Animal Behaviour* **39,** 42–54 (1990).
18. Grosberg, R. K. & Quinn, J. F. The Genetic-Control and Consequences of Kin Recognition by the Larvae of a Colonial Marine Invertebrate. *Nature* **322,** 456–459 (1986).
19. Hamilton, W. D. The genetical evolution of social behaviour. I & II. *Journal of Theoretical Biology* **7,** 1–52 (1964).
20. Dawkins, R. *The Selfish Gene*. (Oxford University Press, 1976).
21. Grafen, A. A geometric view of relatedness. *Oxford surveys in evolutionary biology* **262,** 391–397 (1985).
